# Study protocol for the Australian autism biobank: an international resource to advance autism discovery research

**DOI:** 10.1186/s12887-018-1255-z

**Published:** 2018-08-27

**Authors:** Gail A. Alvares, Paul A. Dawson, Cheryl Dissanayake, Valsamma Eapen, Jacob Gratten, Rachel Grove, Anjali Henders, Helen Heussler, Lauren Lawson, Anne Masi, Emma Raymond, Felicity Rose, Leanne Wallace, Naomi R. Wray, Andrew J. O. Whitehouse, the Australian Autism Biobank team, Jolene Berry, Vandhana Bharti, Dominique Cleary, Melanie De Jong, Mira Frenk, Maryam Haghiran, Alexis Harun, Helen Holdsworth, Anna Hunt, Rachel Jellett, Feroza Khan, Deborah Lennon, Jodie Leslie, Tiana McLaren, Candice Michael, Melanie Muniandy, Melissa Neylan, Michaela Nothard

**Affiliations:** 1 Cooperative Research Centre for Living with Autism (Autism CRC), Long Pocket, Brisbane, QLD Australia; 2 Telethon Kids Institute, University of Western Australia, Perth, WA Australia; 3 Mater Research Institute, The University of Queensland, Brisbane, QLD Australia; 4 Olga Tennison Autism Research Centre, La Trobe University, Melbourne, VIC Australia; 5 School of Psychiatry, University of New South Wales, Sydney, NSW Australia; 6 Academic Unit of Child Psychiatry South West Sydney, Ingham Institute, Liverpool Hospital, Sydney, NSW Australia; 7 Institute for Molecular Bioscience, The University of Queensland, Brisbane, QLD Australia; 8 Wesley Medical Research, Brisbane, QLD Australia; 9 Queensland Brain Institute, The University of Queensland, Brisbane, QLD Australia

**Keywords:** Study protocol, Autism spectrum disorder, Genetic, Genomic, Biobank

## Abstract

**Background:**

The phenotypic and genetic heterogeneity of autism spectrum disorder (ASD) presents considerable challenges in understanding etiological pathways, selecting effective therapies, providing genetic counselling, and predicting clinical outcomes. With advances in genetic and biological research alongside rapid-pace technological innovations, there is an increasing imperative to access large, representative, and diverse cohorts to advance knowledge of ASD. To date, there has not been any single collective effort towards a similar resource in Australia, which has its own unique ethnic and cultural diversity. The Australian Autism Biobank was initiated by the Cooperative Research Centre for Living with Autism (Autism CRC) to establish a large-scale repository of biological samples and detailed clinical information about children diagnosed with ASD to facilitate future discovery research.

**Methods:**

The primary group of participants were children with a confirmed diagnosis of ASD, aged between 2 and 17 years, recruited through four sites in Australia. No exclusion criteria regarding language level, cognitive ability, or comorbid conditions were applied to ensure a representative cohort was recruited. Both biological parents and siblings were invited to participate, along with children without a diagnosis of ASD, and children who had been queried for an ASD diagnosis but did not meet diagnostic criteria. All children completed cognitive assessments, with probands and parents completing additional assessments measuring ASD symptomatology. Parents completed questionnaires about their child’s medical history and early development. Physical measurements and biological samples (blood, stool, urine, and hair) were collected from children, and physical measurements and blood samples were collected from parents. Samples were sent to a central processing site and placed into long-term storage.

**Discussion:**

The establishment of this biobank is a valuable international resource incorporating detailed clinical and biological information that will help accelerate the pace of ASD discovery research. Recruitment into this study has also supported the feasibility of large-scale biological sample collection in children diagnosed with ASD with comprehensive phenotyping across a wide range of ages, intellectual abilities, and levels of adaptive functioning. This biological and clinical resource will be open to data access requests from national and international researchers to support future discovery research that will benefit the autistic community.

## Background

Autism spectrum disorder (ASD) refers to a group of complex and heterogeneous neurodevelopmental conditions behaviourally defined by difficulties in social communication, as well as restricted ranges of interests and/or stereotypic/sensory behaviours [1]. Current prevalence estimates range between 1 and 1.7% of most surveyed populations [2–6], with males more commonly diagnosed than females [7, 8]. Prevalence has risen sharply in the last two decades, attributed largely to increasing awareness, changes in diagnostic criteria, and increased diagnoses of individuals with less severe symptom presentations [4, 9]. More than 70% of individuals diagnosed with ASD will also be diagnosed with a medical (e.g. gastrointestinal, sleep, metabolic condition) or psychiatric (e.g. depression, anxiety) condition across their lifetime [10–12]. Estimated cognitive functioning and language levels also vary considerably across individuals [13, 14].

The highly diverse phenotypic presentation in ASD is also reflected in etiological heterogeneity, involving a combination of genetic and environmental contributors. Early research identifying specific genes responsible for syndromes highly comorbid with ASD, such as fragile X [15] and Rett syndrome [16], supported the role of de novo variations of major effect. Studies of recurrence risk in twin and family studies have also strongly supported the existence of heritable and polygenic risk factors. Heritability estimates, for example, have recently converged at 83%, although significant variability in these estimates have been reported [17–20]. Sequencing studies have provided further support for the role of de novo copy number variants, with increased rates in individuals diagnosed with ASD, and their siblings, compared to individuals without ASD. Replicated gene discovery findings have started to converge on those genes involved in regulation of early development and in synaptic function [21]. It is also becoming increasingly clear that these mutations appear amongst a background of higher frequency of common variants that may account for a significant proportion of liability in individuals diagnosed with ASD. Converging evidence in this area supports ASD as a complex polygenic condition with both de novo and rare inherited variants acting amongst a background of common genetic variation [22, 23].

Large and significantly collaborative bio-resources are required to conduct discovery research, many of which have been established within the last decade [24, 25]. A few ASD-specific biobanks have been created, notably in the USA and across several European countries, resulting in significant and valuable advances in the field. The Simons Foundation Autism Research Initiative (SFARI; including the Simons Simplex Collection [26], Simons Variation in Individuals Project [27], and Simons Foundation Powering Autism Research for Knowledge, SPARK), the Autism Genetic Research Exchange (AGRE; [28]), MSSNG, and the EU-AIMS Longitudinal European Autism Project [29], are amongst the largest resources that have been created, supporting genetic and biological research to inform both diagnostic and treatment discoveries. Alongside these collaborative efforts have been rapid-pace advances in genomic technology, such as high throughput genome sequencing and advances in bioinformatic methods, that have facilitated analysis incorporating thousands of individuals (e.g. [30]), as well as providing a mechanism to identify extremely rare mutations or conditions.

Despite significant support from the autistic community for these large-scale genetic research efforts [31], to date there has not been any attempt to create such a resource in the Australian context. Australia comprises a similar range of socio-demographic variables relative to other international ASD biobanks, but with a unique cultural and ethnic diversity. In addition, many current ASD resources have specific inclusion criteria pertaining to age, verbal or cognitive ability (excluding minimally verbal individuals or those with comorbid intellectual disability), or family history (the Simon Simplex Collection, for example, is restricted to families with only one known individual diagnosed with ASD), limiting their potential generalisability.

In 2013, the Cooperative Research Centre for Living with Autism (Autism CRC; https://www.autismcrc.com.au/) was established and is the world’s first national, cooperative research effort focused on autism across the lifespan. With the support of the Autism CRC, the Australian Autism Biobank was created to house a large repository of detailed phenotypic (observational and reported clinical features) and biological information from a broadly diverse and representative cohort of children with ASD and their families. This repository was later expanded to include several comparison groups, including siblings of probands who do not have a diagnosis of ASD, children recruited from the general community without a diagnosis of ASD, and children who had been clinically queried for an ASD diagnosis, but who did not meet formal diagnostic criteria. Data collection ceased on June 30th, 2018. The aim of this protocol is to describe the study design and data collection methods to support data access requests from national and international researchers. The long-term aim in establishing this biobank was to develop a detailed biological and clinical resource to significantly accelerate discovery genetic and biological ASD research that will support earlier and more accurate diagnostic efforts and facilitate more precise and tailored interventions.

## Methods/design

### Participants

The Australian Autism Biobank comprises four participant groups of children between two to 17 years of age: (i) children diagnosed with ASD (‘ASD probands’); (ii) children queried for ASD but who have not met DSM-5 diagnostic criteria for ASD (‘ASD-Query’); (iii) siblings of children with ASD without an ASD diagnosis (‘siblings’); and (iv) children without a diagnosis of ASD and no first-degree relative diagnosed with ASD (‘controls’). Where possible, both biological parents of ASD probands/ASD-query children were invited to participate to obtain complete family trios. A parent/primary caregiver (hereafter referred to as ‘parent’, but including non-biological parents, grandparents, or foster carers) was required to provide written informed consent for all children to participate in the study; children above the age of 7 years could additionally provide written or verbal assent to participate if their parent deemed them cognitively able to understand the study requirements.

Probands had received a clinically confirmed diagnosis of ASD per DSM-IV [32] or DSM-5 [1] criteria, depending on their age at diagnosis. A participant group was also created to include children who had been queried by a health professional for an ASD diagnosis, but did not reach DSM-5 criteria for ASD, and who may also be siblings of probands (‘ASD-Query’ participants). All children with a diagnosis of ASD within a family were invited to participate, where possible, including full and half siblings and concordant/discordant twins. No exclusion criteria were applied with respect to conditions other than ASD (for example, other psychiatric, medical or genetic conditions), cognitive function level, or medication use. For all participants, sufficient English to provide written informed consent (from parents) or English spoken at home (for children) was required.

### Settings

Participants were recruited through four sites/states in Australia: (1) the Telethon Kids Institute, University of Western Australia (Perth, Western Australia); (2) the Olga Tennison Autism Research Centre, La Trobe University (Melbourne, Victoria); (3) the University of New South Wales (Sydney, New South Wales); and (4) Lady Cilento Children’s Hospital (LCCH, Brisbane, Queensland). Each site comprised of a principal research investigator, a postdoctoral researcher and/or trained research officers for data collection. Phenotypic and biological data collection was conducted in clinical facilities at each site. Biological samples were processed at the Institute for Molecular Bioscience, University of Queensland (Brisbane, Queensland), and stored in long-term biobanking facilities. All phenotype data collected on record forms were converted to an electronic format, audited, and stored centrally.

Ethical approval for this study was provided by human research ethics committees at Princess Margaret Hospital for Children (2014029EP), La Trobe University (HEC16–104), Sydney Children’s Hospital Network (14/SCHN/269), Mater Health Services (14/MHS/212), the University of Queensland (2014001079), and the University of Western Australia (RA/4/1/8184).

### Procedures

**Fig. 1:**
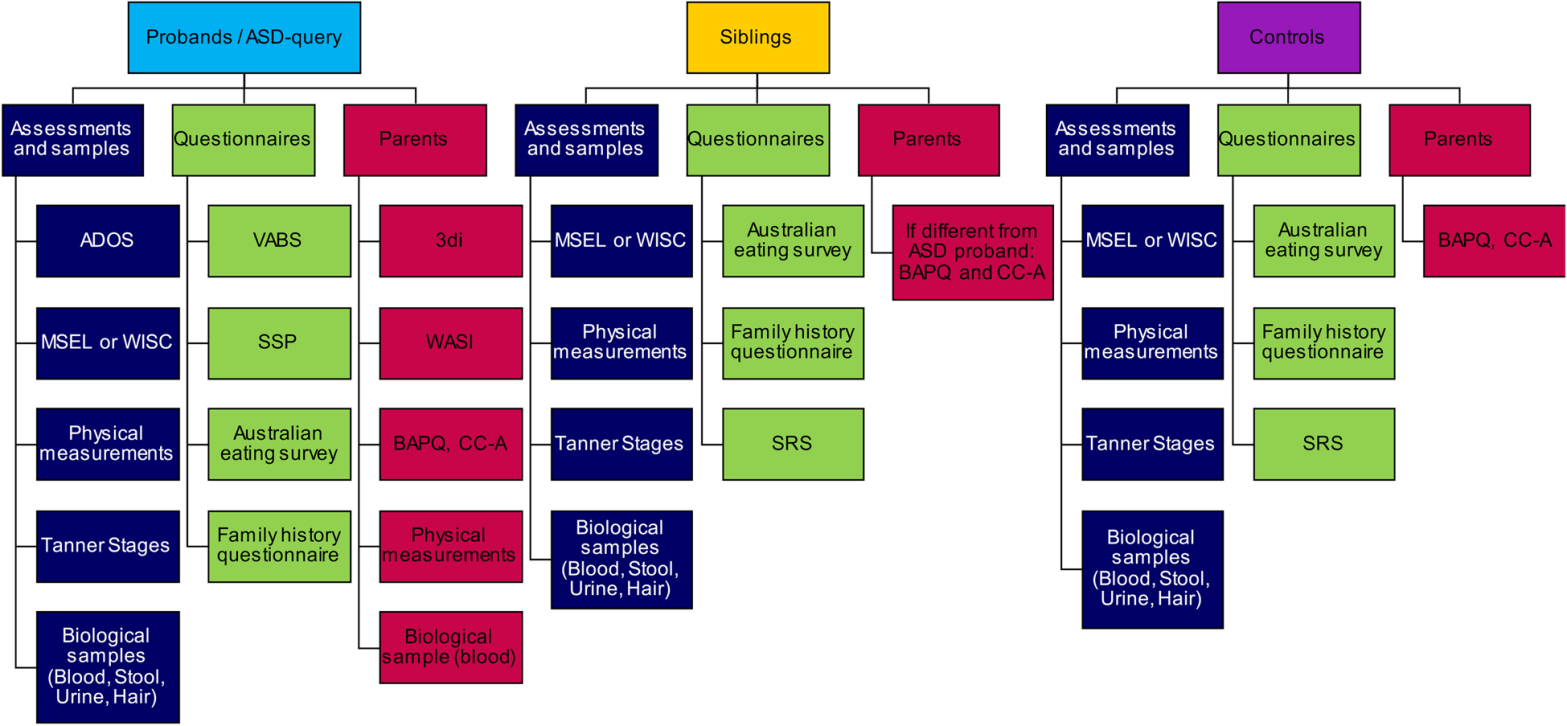
Summary of data collected by participant group. *Abbreviations*. ADOS: Autism Diagnostic Observation Schedule, BAPQ: Broader Autism Phenotype Questionnaire, CC-A: Communication Checklist – Adult, MSEL: Mullen Scales of Early Learning, SRS: Social Responsiveness Scale, SSP: Short Sensory Profile, WASI: Wechsler Abbreviated Scale of Intelligence, WISC: Wechsler Intelligence Scale for Children

Standardised clinical assessments were conducted with each child, including both parents where possible, and involving a parent to facilitate completion of clinical assessments with children where necessary. A reduced set of these measures was obtained from siblings and controls; see Fig. 1 for an overview of data collected from each participant group.

Biological sample collection was attempted for all children. As collection of blood samples can be distressing for children, individual sites created tailored social stories to facilitate understanding of the procedures involved. Tailored instructions were also created for parents to support collection of stool, urine, and hair at home, for both trained and non-toilet trained children. For unsuccessful home collections, a second collection of these samples was attempted at the clinical appointment, where possible. Blood, stool, and hair were immediately shipped to the University of Queensland’s Institute for Molecular Bioscience for initial processing, labelling, and transfer to the biobanking facilities at Wesley Medical Research. Urine samples were kept frozen at each site and periodically shipped in batches on dry ice to the University of Queensland’s Institute for Molecular Bioscience for labelling and then transferred to long-term storage.

Clinical assessments and blood sample collections were ideally conducted during one appointment, with parents mailed out questionnaires and sample collection kits prior to this appointment. In some cases, assessments were split across multiple appointments, particularly for families with several children participating, those who were recruited as part of other research studies within data collection sites, or where children were diagnosed at different times to their siblings. Research staff attempted to follow up any missing data through phone calls or emails to families. All clinical assessments and questionnaires were checked and scored by individual sites. Reliability between research staff on clinical assessments was maintained by each site. All hardcopy de-identified questionnaires and assessments were entered via a web-portal hosted on central servers.

### Biological samples

#### Blood

Venous blood samples were collected by trained paediatric phlebotomists or through hospital/pathology phlebotomy services. Samples were then transported at room temperature to the University of Queensland’s Institute for Molecular Bioscience and immediately processed (time from collection to processing between 12 and 72 h). EDTA (for DNA), SST (for serum), and PAXgene (for RNA) tubes were used to collect blood from children and parents (due to processing requirements, PAXgene samples are only available for a selected number of families). Collection of whole blood into EDTA tubes was prioritised for the purposes of obtaining DNA. Where a blood collection was unsuccessful (due to child distress, difficulty obtaining a sample, or withdrawal of parental consent), a saliva collection (2 ml through spit or swab) was attempted.

Whole blood collected in EDTA or SST tubes were stored at room temperature during transportation and then centrifuged at 3000 rpm for 15 min to separate the individual components of plasma, red blood cells, buffy coat (EDTA) and serum (SST). Plasma, red blood cells and serum were manually pipetted into 2 mL screw cap tubes for long-term storage at − 80 degrees Celsius. Buffy coats were added to a 50 ml tube containing 25 mL 1 x TE (ph 8) and gently inverted before re-centrifugation for a further 10 mins at 3000 rpm. This washing step lyses contaminating red blood cells that have been collected with the buffy coat to effectively remove exposed haemoglobin; known to interfere with the quality of extracted genomic DNA.

#### Stool

Parents chose a method of stool collection best suited for their child’s toileting level, either collected from a liner suspended in a toilet bowl or scraped from diapers. Two individual teaspoon stool samples were collected and suspended in 4mLs RNAlater™. Samples were transported to the University of Queensland’s Institute for Molecular Bioscience and immediately processed (time from shipping to processing 12–72 h). Each stool sample was vigorously homogenised before being aliquotted into 3 × 1 mL samples for long-term storage at − 80 degrees Celsius.

#### Urine

Parents chose a method of urine collection best suited for their child’s toileting level, either as a mid-stream collection, by a pipette from a toilet liner suspended in a toilet bowl, from cotton balls placed in a nappy, or by using a paediatric urine collection bag. Parents were instructed to attempt to collect the first urination of the morning, where possible, and freeze samples immediately. These frozen samples were then transported, with frozen ice packs used to maintain temperature, by families when attending their clinical assessments at each site. Upon receipt, samples were immediately transferred to a − 80 °C freezer, noting condition of sample upon receipt (frozen/not frozen, partially thawed, etc). Samples were transferred in batches to the University of Queensland’s Institute for Molecular Bioscience for labelling and then transferred to long-term storage.

#### Hair

Hair samples, approximately 10 strands, were collected at the base of the head, cut close to the scalp, without the hair follicle. Samples were placed on aluminium foil and transported at room temperature to the University of Queensland’s Institute for Molecular Bioscience for labelling, and then transferred to long-term storage.

See Table 1 for a summary of the biological samples collected from each participant group.

**Table 1:** Biological samples collected by participant group

	ASD-Probands/ASD-Query	Parents	Siblings/Controls
Blood^a^			
EDTA	6 ml	10 ml	6 ml
SST	5 ml	5 ml	5 ml
PAXgene^b^	2.5 ml	2.5 ml	2.5 ml
Stool^c^	2 × 1 teaspoon	–	2 × 1 teaspoon
Urine^c^	~ 20 ml	–	~ 20 ml
Hair^c^	~ 10 strands	–	~ 10 strands

^a^Saliva collection was attempted where a blood sample collection was unsuccessful; ^b^PAXgene samples (for RNA) collected on a sub-set of participants; ^c^Stool, urine, and hair collected on a sub-set of children

### Clinical phenotyping

#### ASD probands/ASD-query

##### ASD symptoms

The Autism Diagnostic Observation Schedule-2 (ADOS; [33]) is a semi-structured standardised observational assessment designed to elicit social-communication and repetitive behaviours relevant to an ASD diagnosis, administered by researchers who had obtained research-reliable coding. The most appropriate ADOS module was administered, based on the participant’s age and current expressive language ability: Module 1 (pre-verbal children with single words/simple phrases), Module 2 (children with flexible phrase speech), Module 3 (children or adolescents with fluent language), and Module 4 (older adolescents with fluent language). Behaviours are coded across five domains: language and communication, reciprocal social interaction, play, stereotyped behaviours and restricted interests, and any other abnormal behaviours. Codes are then scored to an algorithm to derive a standardised comparison score across modules, relative to age and/or language ability; higher scores are indicative of greater ASD traits.

The Developmental, Dimensional, and Diagnostic Interview (3di; [34]) is a computerised semi-structured interview designed to support ASD diagnostic interviews with minimal training and ongoing support. It was developed based on Autism Diagnostic Interview-Revised (ADI-R; [35]) question formats and algorithm-based symptom analysis with additional DSM-5 symptom subscales. Interview questions relate to medical history, language and non-verbal communication, play and friendships, reciprocal social interaction, repetitive behaviours and restricted interests, along with optional sections on relevant childhood comorbidities. The interview can be partially completed through questionnaires or completed through an interview. The interview yields both quantitative severity scores on diagnostic criterion as well as number of criterion met.

##### Cognitive function

Two cognitive measures were administered, based on children’s chronological age. For children between 2 and 6 years of age, the Mullen Scales of Early Learning (MSEL; [36]) was used, which is a standardised developmental assessment that incorporates interactive and play-based tasks. Four domains were assessed: Fine Motor, Visual Reception, Expressive Language, and Receptive Language. These four scales were summed to yield an Early Learning Composite Score, as an estimate of cognitive functioning (M = 100, SD = 15). For each domain, raw scores, a corresponding T-score, percentile rank, and an age equivalent, was recorded. For children aged above 6 years, the Wechsler Intelligence Scale for Children (WISC, 4th edition; [37]) was used, a standardised measure of cognitive functioning. Ten structured activities elicit cognitive abilities in four domains: Verbal Comprehension, Perceptual Reasoning, Working Memory, and Processing Speed. These four domains sum to give a Full-Scale IQ estimate (M = 100, SD = 15). Raw and scaled scores (relative to chronological age) were recorded for each activity, along with summed scaled scores, composite scores, percentile rank and 95% confidence intervals for each domain.

##### Questionnaires

As a measure of adaptive behaviour, the Vineland Adaptive Behavior Scale was administered (VABS, 2nd edition; [38]). This standardised and norm-referenced measure was completed as a parent questionnaire that assesses four domains of adaptive behaviour: Communication, Daily Living Skills, Socialization, Motor Skills (for children under 6 years of age), that sum together giving an Adaptive Behavior Composite score. Raw and scaled scores, percentile ranks, and age-equivalents, were recorded for each domain and sub-domain. Maladaptive behaviours were also assessed, yielding a summary score of internalizing, externalizing, and other maladaptive behaviours.

The Short Sensory Profile (SSP, 2nd edition; [39]) is a 34-item parent-reported measure of behavioural sensory processing that assess difficulties with processing and responding to sensory input. Parents rated a range of behaviours on a scale of 1 (almost never) to 5 (almost always), which sum to four subscales (Sensory Seeking, Avoiding, Sensitivity, Registration) and two scales of Sensory and Behavioural scores. Raw scores, percentile ranks, and rank relative to a normal distribution is calculated.

The Children’s Communication Checklist (CCC, [40]) is a 70-item parent-reported measure that assesses communication impairments relevant to both specific language disorders and their overlap with ASD. The CCC was completed as part of the 3di, administered either as a questionnaire or during the interview. Items are completed on a four-point scale (no, does not apply; applies somewhat; definitely applies; unable to judge) that sum to nine subscales related to pragmatic language skills that are necessary for social communication. Five of these scales sum to a pragmatic composite score. Items were omitted for children with current expressive language comprising only single words, and the questionnaire is not completed for children who are minimally verbal or have no spoken language.

For any stool sample collections, parents additionally completed the Australian Child and Adolescent Eating Survey [41] to assess food frequency, usual food habits, and nutrient intake over the previous six months. Energy (kJ) and nutrient (protein, fat, carbohydrates, sugars, fibre etc) intake was calculated based on parental reports.

A bespoke family history questionnaire was designed to capture medical history, pregnancy related factors (e.g. stress or complications), early childhood development, diagnostic history, current medication, sleep and gastrointestinal function, as well as parental demographics and health.

##### Measurements

Physical development was measured by height, weight, and head circumference measurements. The Tanner Stages [42, 43] was used to determine physical pubertal development, where parents selected descriptions of physical characteristics (genital, breast, pubic hair development) to rate approximate pubertal stage for children 8 years of age and above. Researchers conducting the clinical assessment also assessed any overt physical anomalies (fingers/hands, neck and spine) and provided comments on behaviour during the clinical assessment.

#### Siblings/controls

##### ASD symptoms

The Social Responsiveness Scale (SRS, 2nd edition; [44]) is a questionnaire completed by parents about autistic symptomology in children. It provides a quantitative measure of autistic traits in clinical and non-clinical samples and exhibits good reliability compared to more comprehensive ASD diagnostic measures, such as the ADI-R [45]. The SRS is a 65-item questionnaire rated on a 0 (never true) to 3 (almost always true) scale that yields five subscales (social awareness, social cognition, social communication, social motivation, restricted and repetitive behaviours) and a total score. The questionnaire also yields two DSM-5 relevant subscales (social communication, restricted and repetitive behaviours). Both raw and scaled T-scores were calculated.

##### Cognitive function

Based on the child’s age, the MSEL [36] or the WISC 4th edition [37] was administered to obtain a standardised measure of cognitive functioning.

##### Other questionnaires

Parents completed the Australian Child and Adolescent Eating Survey [41] once a stool sample was collected. A family history questionnaire, described above, was also completed.

##### Measurements

The same physical measurements and clinical ratings were also collected on siblings/controls.

#### Parents of ASD probands or ASD-query children

##### ASD symptoms

The Broad Autism Phenotype Questionnaire (BAPQ; [46]) elicits personality and language characteristics related to the ASD phenotype in parents of individuals diagnosed with ASD. Thirty-six items are self-reported on a 6-point scale (from very rarely applies to applies very often) and summed to three subscales (social behaviour, stereotyped-repetitive behaviour, and communication).

##### Cognitive function

The Matrix Reasoning subtest of the Wechsler Abbreviated Scale of Intelligence (WASI, 2nd edition; [47]) was used as a measure of nonverbal reasoning.

##### Other questionnaires

The Communication Checklist – Adult (CC-A; [48]) was developed as an adult extension of the 2nd edition of the CCC [49] that assesses communicative behaviour. It is completed by an informant who knows the individual well, in this case usually the other person’s partner and/or parent of the child with ASD. The questionnaire includes three subscales related to language structure, pragmatic skills, and social engagement. Previous research indicates the CC-A may be sensitive in assessing the broader autism phenotype in parents of children with ASD [50].

##### Measurements

Height, weight, and head circumference were also collected on parents at the time of blood collection.

#### Parents of siblings or controls

For parents of siblings who were not biologically related to the ASD proband(s), the BAPQ was also completed, where possible. For parents of controls, both the BAPQ and CC-A were completed.

### Database

All phenotypic (observational and parent-reported) data was collected on hard copy record forms and entered remotely by each data collection site into databases hosted centrally by Wesley Medical Research. Data officers performed a 100% audit on all data entered for accuracy against scanned copies of de-identified record forms.

### Access

Ongoing management of this study is overseen by an Operations Committee. Access to data can be requested through an application to a Data Access Committee. This committee includes representatives from the Autism CRC, researchers, and the autistic community. This committee reviews any applications for phenotypic and/or biospecimens stored in this biobank in compliance with guidelines issued by the National Health and Medical Research Council (NHMRC) under the *National Health and Medical Research Council Act 1992* and guidance from the Autism CRC Board. Access to data is subject to scientific review and a data access fee.

## Discussion

Significant and rapid progress has been made into the complex genetic, biological and interacting environmental mechanisms contributing to an ASD phenotype. Alongside such advancements is the imperative for large and well-characterised cohorts of participants diagnosed with ASD from diverse and representative families. Several successful international biobanks have supported many scientific discoveries in this area to date, but have collected limited amounts of clinical information, restricted sample collection to saliva samples to favour large-scale collection, or have imposed restrictive inclusion criteria to allow for more homogenous participant groups.

The Australian Autism Biobank is the first effort of its kind in Australia, designed to overcome these previous limitations by establishing an international resource that will facilitate effective and timely research in this area. While this biobank may not form the largest collection of biological samples in ASD research, it is the only resource we are aware of to collect multiple biological specimens alongside detailed ‘deep’ clinical phenotyping [51] in a diverse sample. This data is being collected from families with at least one child diagnosed with ASD, incorporating a diverse range of clinical phenotypes, as well as language and cognitive abilities, across the childhood age range. Previous attempts to collect blood for ASD genetic research alongside stool, urine, or hair have been in much smaller numbers of selected samples; the creation of this biobank provides, for the first time, the opportunity to ask very detailed questions about the potential interactions between genetic and biological (metabolic, gastrointestinal, and other) mechanisms underlying ASD. This biobank also specified a minimal set of inclusion criteria, to allow for children with a range of language and cognitive abilities to participate; minimally verbal children, or those with comorbid intellectual disability, are often under represented or are explicitly excluded participant groups in many research protocols involving the collection of biological samples. By keeping the inclusion criteria broad, this biobank allows a unique diverse and representative sample cohort to support novel and replication research questions that allows for the known heterogeneity of ASD to be represented.

The addition of sibling and control comparison samples allows researchers to account for similar environments and shared genetic pathways. A significant number of probands participating in this biobank are also part of complex families with multiple children or relatives diagnosed with ASD. For example, much of our current understanding about ASD genetics comes from the analysis and careful selection of families with ‘simplex’ histories (an absence of ASD diagnoses in first-degree relatives); the inclusion of large and complex multiplex families allows for the examination of novel and testable hypotheses about inherited genetic variations of smaller effect [52]. The collective value of this resource will not just be in the discovery of potential novel genetic findings, but in the ability to combine samples with other research groups to support essential replication efforts to move the field forward.

To facilitate the success of this biobank, and ensure a diverse and representative cohort was achieved, relationships between individual data collection sites and relevant health practitioners or organisations were established, including paediatricians, clinical psychologists, speech pathologists, diagnostic clinics, intervention services, and disability organisations. Efforts were also made to invite families from regional or remote areas of Australia to participate where possible; for example, by contributing only questionnaires and saliva samples via mail, or by conducting clinical assessments during weekends.

Practical considerations were made over the course of the study that facilitated the completion of our aims. One such step was to ensure protocols were in place to successfully achieve biological sample collection for most families, including very young children, those with cognitive or language impairments, and those with variability in toileting behaviours. Tailored social stories for blood collection, pictorial collection instructions for stool, urine, and hair samples, and creation of different methods of sample collections based on levels of toilet training, all facilitated better success rates in collections. Other practical considerations included tailoring ID structures, questionnaire administration, and clinical assessments for families, particularly those with multiple children diagnosed with ASD.

In conclusion, the creation of this biobank has resulted in a valuable international resource that will support large-scale novel discovery ASD research. The specific decision to include a diverse sample, with respect to age, cognitive function, language ability, and adaptive function, from ethnically and culturally diverse populations in Australia, has ensured that this resource will be able to ask both novel questions and support replicability of research findings from other established ASD repositories. Major genetic and biological advances have been made over the last decade, and it is our hope that this resource will further extend research in this area. The detailed clinical information accompanying the biological samples also allows for a variety of questions to be asked around clinical presentations, subtypes, and patterns of traits within and across families at different developmental periods. Importantly, the trust and support from families in contributing this data has been vital, ensuring that this resource will remain an important and valued mechanism to support future discoveries that will benefit the autistic community.

## Abbreviations

3di: Developmental, Dimensional, and Diagnostic Interview
ADI-R: Autism Diagnostic Interview-Revised
ADOS: Autism Diagnostic Observation Schedule
ASD: Autism Spectrum Disorder
Autism CRC: Cooperative Research Centre for Living with Autism
BAPQ: Broader Autism Phenotype Questionnaire
CC-A: Communication Checklist – Adult
CCC: Children’s Communication Checklist
DSM-5: Diagnostic and Statistical Manual of Mental Disorders, Fifth Edition
DSM-IV: Diagnostic and Statistical Manual of Mental Disorders, Fourth Edition
EDTA: Ethylenediaminetetraacetic acid
MSEL: Mullen Scales of Early Learning
SRS: Social Responsiveness Scale
SSP: Short Sensory Profile
SST: Serum-Separating Tube
VABS: Vineland Adaptive Behaviour Scale
WASI: Wechsler Abbreviated Scale of Intelligence
WISC: Wechsler Intelligence Scale for Children
